# The Impact of Legal Coercion on the Therapeutic Relationship in Adult Schizophrenia Patients

**DOI:** 10.1371/journal.pone.0124043

**Published:** 2015-04-24

**Authors:** Friederike X. E. Höfer, Elmar Habermeyer, Andreas Mokros, Steffen Lau, Stefanie K. Gairing

**Affiliations:** 1 Department of Forensic Psychiatry, University Hospital of Psychiatry, University of Zurich, Rheinau, Switzerland; 2 Department of General Psychiatry, University Hospital of Psychiatry, University of Basel, Basel, Switzerland; Benito Menni Complejo Asistencial en Salud Mental, SPAIN

## Abstract

The quality of the therapeutic relationship between psychiatric patients and their attending physicians plays a key role in treatment success. We hypothesize that mandatory treatment is negatively associated with the quality of the therapeutic relationship. In a cross-sectional study design, data on psychopathological symptom load (as captured with the Brief Psychiatric Rating Scale) and on the quality of the therapeutic relationship (as measured with the Scale to Assess the Therapeutic Relationship) were collected from 113 adult male psychiatric patients and 35 attending physicians. Patients belonged to one of three groups: self-referred or involuntarily admitted patients from general psychiatry wards or patients from medium secure forensic psychiatric units. On average, self-referred patients rated the quality of the therapeutic relationship significantly more positive than did involuntarily admitted patients in general psychiatry wards. Forensic psychiatric patients, on average, gave an intermediate rating of the quality of the therapeutic relationship. There was no association between patients’ ratings and physicians’ ratings of the quality of the therapeutic relationship. Patients’ ratings of the quality of the therapeutic relationship were inversely related to symptom severity in general and hostility in particular. Ratings of the quality of the therapeutic relationship are not associated with patients’ legal status but rather with patients’ symptoms of hostility.

## Introduction

The therapeutic relationship (TR) has recently been described as an important process factor in psychiatry and psychotherapy. The TR is widely recognized as playing a key role in treatment adherence [1], symptom reduction, medication adherence [2, 3], outcome of psychosis and psychotherapy treatment [3–5], quality of life [6], and accurate correct evaluation of psychiatric patients’ risk of violence [7]. The quality of the TR also has positive effects on patient satisfaction, personal trust [8], and interventions to prevent criminal behavior. Several studies have shown an association between the quality of the TR and lower dropout rates, better medication adherence, fewer readmissions, and improved symptom levels for patients suffering from schizophrenia [9–11].

Coercive measures in psychiatry are assumed to handicap, or even worse to erode, the TR [12]. Previous authors have described the lack of agreement on the tasks and goals of therapy as a major contributing factor to the breakdown in the TR [13]. These aspects become even more relevant in forensic psychiatry where the legal basis leads to highly restricted treatment settings. Hence, the use of legal coercion to reduce the risk of delinquency of mentally ill criminal offenders can be expected to influence the TR negatively. The conflicting role of therapists in mandatory treatment settings—with care on the one hand and special requirements imposed by judicial orders on the other—has been widely debated, representing a dilemma between maximum permissible patient centricity and public safety [14]. Thus, therapists in forensic psychiatry cannot focus exclusively on the patients’ therapeutic needs and demands.

Therapeutic relationships in psychiatry are subject to different legal conditions with varying degrees of coercion. This fact gave rise to our hypothesis that legal coercion is negatively associated with the quality of the TR. Our assumption was that both the patient and the attending physician would rate the quality of the TR more negatively in forensic psychiatry than in general psychiatry. The reason for this assumption is that in forensic psychiatry, legal coercion limits the patients’ freedom more severely and over a longer period of time. To the best of our knowledge, no study has yet compared the quality of the TR in forensic and general psychiatry inpatient services. In the present study, the differential effects of common legal conditions on the TR were examined by comparing forensic psychiatric inpatients with general psychiatric inpatients. The covariates that were associated with the ratings of the quality of the TR were also identified. In particular, the severity of psychopathological symptoms was scrutinized. Finally, the correlations reported thus far between patient and physician judgments of the quality of the TR have tended to be small [9, 15–17], ranging from *r*_s_ = .13 [9] to *r* = .31 [17]. The level of agreement between the views of patients and clinicians on the quality of the TR may at least in part be attributed to measurement error. Therefore, we assessed the relationship between patients’ and clinicians’ evaluations of the quality of the TR by structural equation modelling (SEM), which allows for an error-free estimation of the strength of association between patient and clinician ratings.

## Methods

**Fig 1:**
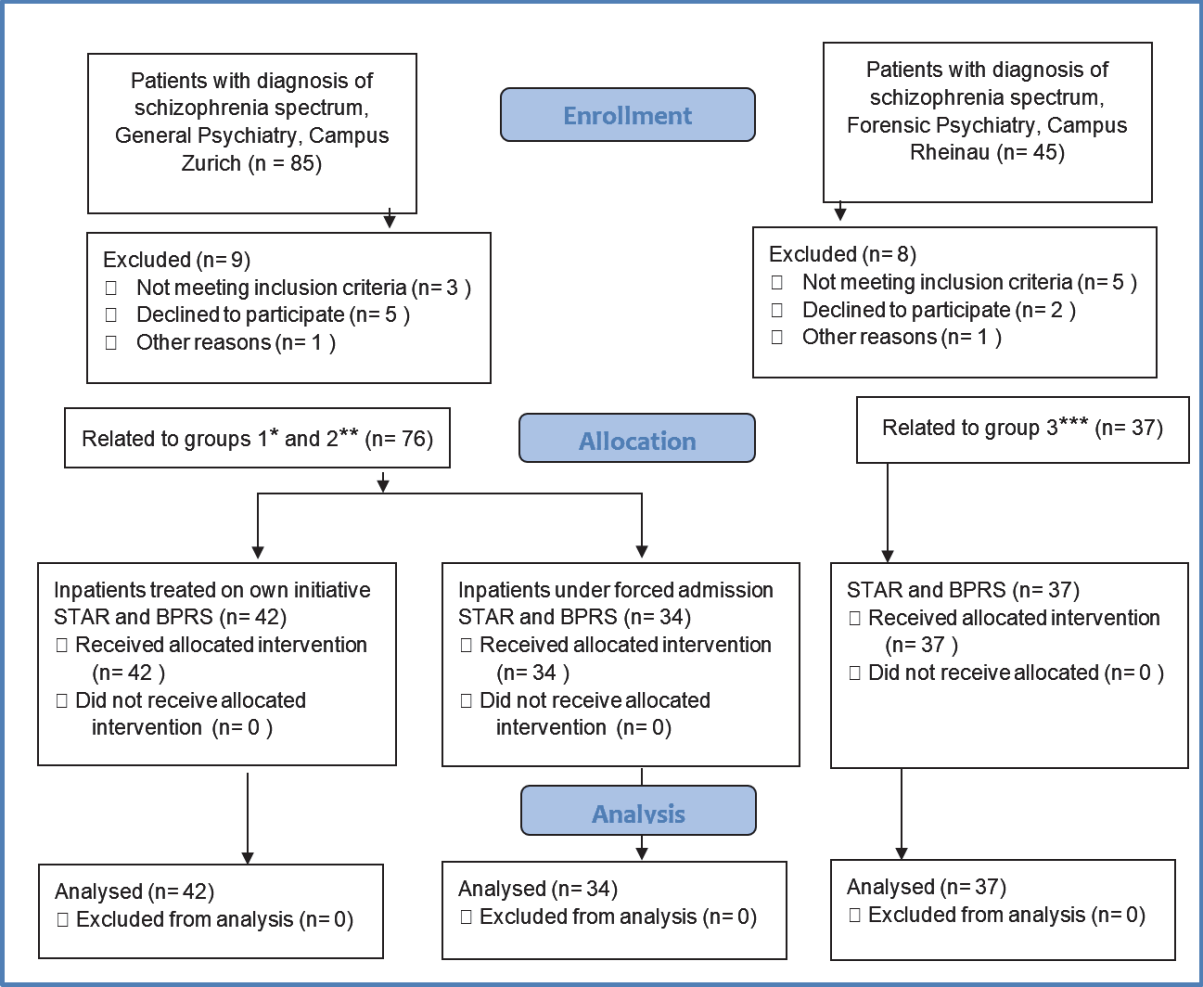
Recruitment. A depiction of the process of enrolment, allocation, and analysis of patients in the three groups: self-referred inpatients, inpatients under involuntary admission, and forensic inpatients.

This cross-sectional study comprised a sample of male patients diagnosed with schizophrenia or schizophrenia-like disorders. (In general psychiatry, the diagnosis was made by each participant’s responsible senior physician; in forensic psychiatry, the diagnosis was made based on a senior physician’s expert opinion.) Between September 2011 and September 2012, 113 male inpatients were recruited in three settings in Switzerland (Fig 1): (1) self-referred inpatients in a general psychiatry department at the University Hospital of Psychiatry Zurich (*n* = 42), (2) inpatients under involuntary admission in civil psychiatry (article 397 ff. at the time of investigation; since January 1, 2013, article 426 ff. Swiss Civil Code) at the University Hospital of Psychiatry Zurich owing to aggressive behavior toward self or others (*n* = 34), and (3) forensic inpatients (*n* = 37) from a medium secure forensic psychiatry unit located at the Centre for Forensic Psychiatry Rheinau. All forensic patients were under mandatory treatment order (article 59 Swiss Penal Code). Each participant gave his written informed consent to participate in this study.

Patients were assigned to one of the three groups based on their legal status (self-referred, involuntary admission, or forensic). A randomized allocation was not feasible owing to the study design. Neither the study participants nor the data collector were blind to the study objectives. To control for confounding factors, groups were matched according to age as well as to years and level of education. Ethical approval was granted by the Kantonale Ethikkommission Zürich, Switzerland (no.2010-0562/3). Demographic data were verified by means of structured interviews and combined with data in the case files. The case report forms captured information on demographic characteristics, previous psychiatric treatment, history of delinquency, admission data, and data of the attending physician.

### Study participants

#### Patients

The inclusion criteria were (1) male sex, (2) age 18 to 45 years, and (3) a primary diagnosis of a schizophrenia spectrum disorder (F20.0-F20.9, F21, F22, or F25) according to the International Classification of Diseases, 10th Revision (ICD-10) [18]. Study participants had to be capable of giving written informed consent to participate in this study. They also had to possess sufficient written and spoken command of the German language and had to have been seen by their attending physician at least three times before the questionnaire was handed out. The study investigator was independent and not involved in the treatment of any of the patients. In addition, the patients were informed that participation in the study would not affect their legal status positively or negatively. Newly hospitalized patients were screened, and those who fulfilled the inclusion criteria were enrolled consecutively in this study. Patients and their attending physicians received information on the study in both oral and written form. If both gave their written informed consent, they were requested to fill out the corresponding German version of the Scale to Assess Therapeutic Relationship (D-STAR) questionnaire (D-STAR-P [patient] or D-STAR-C [clinician]) separately [15, 19]. A clinical interview of approximately 20 minutes in duration was then carried out by the study investigator so as to record information on the psychopathological status of the patients in a standardized way. We obtained medical record files, pre-admission reports, and discharge summaries for each participant.

#### Physicians

In total, 35 physicians (general psychiatry group, *n* = 26; forensic group, *n* = 9) rated 113 patients. In the general psychiatry group, the physicians were at the beginning of their training in psychiatry. In the forensic group, most physicians were more experienced and had received or were in the process of receiving specialist training in the treatment of mentally ill offenders. All physicians were supervised by psychiatric consultants on a daily basis. The principal investigator was not involved in any treatment decision.

### Instruments

#### Symptom assessment

Symptom severity was assessed by the investigator using the German version of the Brief Psychiatric Rating Scale (BPRS) [20, 21]. The BPRS is a well-established observer rating scale and contains 18 items [22]. The BPRS was developed to assess categories of psychiatric symptoms in adult schizophrenic inpatients; 19 categories of psychiatric symptoms are examined. Each symptom is rated on a 7-point scale, ranging from “not present” (1) to “extremely severe” (7). The BPRS total score reflects the degree of mental dysfunction. Moreover, five scales are extractable. The reliability (as estimated by internal consistency through Cronbach’s alpha coefficient) of the total score lies between 0.87 and 0.97 and between 0.52 and 0.90 for the four subscales of (1) negative symptoms, (2) depression/anxiety, (3) hostility/uncooperativeness, and (4) positive symptoms [20].

#### Assessment of the therapeutic relationship

There are several questionnaires available to assess the TR. For the present study, STAR was used because of its psychometric properties and the availability of an established German version [15, 19]. Furthermore, the patient (STAR-P) and clinician (STAR-C) versions of STAR allow assessment of the TR from both perspectives.

Both the patient and clinician versions of STAR consist of 12 items, which in turn load onto three subscales: The subscales labeled “Positive Collaboration” and “Positive Clinician Input” are present in both the patient (STAR-P) and clinician (STAR-C) versions; the third subscale is labeled “Emotional Difficulties” in the clinician version and “Non-Supportive Clinician Input” in the patient version. Each item is rated on a scale between 0 (never) and 4 points (always). The retest reliability of the German version of the STAR-C and STAR-P total scores was estimated at *r* = .68 and *r* = .76 over an interval of 29 days, respectively [15], and can thus be considered adequate.

Data privacy and protection were ensured. All participants filled out the questionnaire in the presence of the same study worker who provided them with explanations if required.

### Data analysis

Data were analyzed using Statistical Product and Service Solutions (SPSS) software, version 20. The type I error rate was set at *p* ≤. 05. To evaluate global differences between the three groups, analysis of variance (ANOVA) and multivariate ANOVA were used; SEM models were estimated using Mplus software, version 6.

## Results

One hundred thirteen study participants were assessed in a cross-sectional study using a consecutive sample of patients admitted to eight acute psychiatric units and four medium secure forensic psychiatry units. Data included 113 patient (STAR-P, BPRS) and 113 physician (STAR-C) ratings from 35 physicians. No case with missing data on more than 10% of items was observed. Missing values were not imputed. Instead, average scores were computed based on the items with valid answers.

### Group characteristics

#### Participants

Half of the 113 patients (51%) were of Swiss nationality, and 24.6% originated from non-European countries. The average age of all patients was 33.35 years (*SD* = 9.18). There were no significant group differences in terms of mean age, years and level of education, medication dose, age of onset, age of first criminal offense, number of admissions, and number of months/years spent in psychiatric services (Table 1). All patients had experienced both psychiatric inpatient and outpatient treatment previously, including former experiences of involuntary admissions. The majority of patients (86%) had no current partner, and 5% described their attending physicians as an “important other.” All were undergoing treatment with antipsychotic medication, with most patients being administered two or more drugs. Even though the involuntary group consisted of patients who had been acutely admitted to hospital for psychosis, none of the patients was acutely psychotic at the time of assessment because we waited until patients were receiving sufficient medication. According to their records, all patients included in this study had a prior criminal record. The forensic patients’ mandatory treatment orders had been issued for property, violent, and sexual offenses (e.g., theft, murder, rape, and arson). The three groups differed in treatment duration: 39 days for the self-referred group, 29 days for the involuntary admission group, and 219 days for the forensic group. There was no significant correlation between treatment duration and ratings of the TR based on either STAR-P (*r* = .07) or STAR-C (*r* = .05).

**Table 1:** General characteristics of the self-referred, involuntary admission, and forensic groups.

	Self-referred group (*n* = 42)	Involuntary admission group (*n* = 34)	Forensic group (*n* = 37)	Total sample (*n* = 113)
	Mean	Mean	Mean	Mean
**Age, years *F*(2,110) = 2.70, *p* = .07)**	31.90	32.06	36.19	33.35
**Age of first psychiatric admission, years *F*(2,105) = 2.23, *p* = .11)**	23.90	21.45	25.39	23.61
**Previous criminal offences, number *F*(2, 36) = 5.502, *p* = .00)**	1.00	10.38	2.95	3.92

#### Physicians

In general psychiatry, physicians (*n* = 26, *n*_self-referred_ = 13; *n*_involuntary admission_ = 13) were predominately native speakers of the German language (83%), with an average age of 33.67 years (*SD* = 8.34) in the self-referred group and 31.35 years (*SD* = 4.27) in the involuntary admission group. In forensic psychiatry, the average age of the physicians (*n* = 9) was 45.22 years (*SD* = 14.86). Each physician was treating between 8 and 12 inpatients (*M* = 10.1, *SD* = 3.8) at the time of assessment. Some physicians rated more than one patient, and all physicians rated only a few of the patients they treated in total because of the case mix of the patients they were in charge of.

#### Symptoms

The involuntary admission group had a higher BPRS total score (*M* = 36.76, *SD* = 9.60) than the forensic (*M* = 31.78, *SD* = 8.58) and self-referred groups (*M* = 33.50, *SD* = 11.81). However, these differences did not reach statistical significance, *F*(2,110) = 2.18, *p* = .12. There was a statistically significant positive correlation of *r* = −.41 (*p* <. 001) between the BPRS hostility score and the patient rating of the quality of the TR (i.e., STAR-P). By contrast, the corresponding correlation between the BPRS hostility score and the clinician rating of the quality of the TR (i.e., STAR-C) was negligible and statistically nonsignificant (*r* = −.05, *p* = .57). There were no significant differences between the three groups on any of the remaining BPRS subscales, and the scores on these scales showed no dimensional relationship to the rating of the quality of the TR.

#### Therapeutic relationship

**Fig 2:**
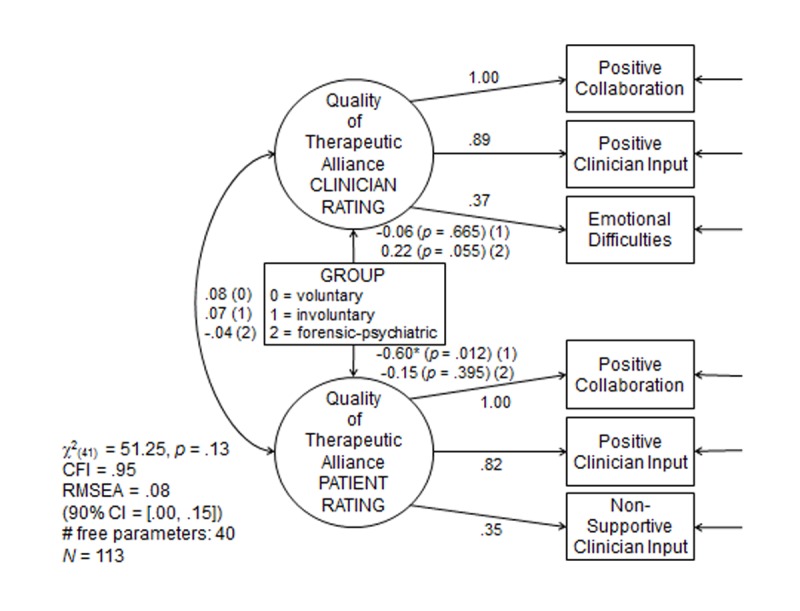
Structural Equation Modeling (SEM). Based on a comparative fit index (CFI) of 0.95 and using the method of power estimation for SEM[23][23][33][33] [33], we estimated the achieved power of the SEM model to be only. 275 only. We used the covariance matrix of the reference group (i.e., the self-referred general psychiatric patients) as a basis for the estimation of achieved power.

Following the conceptualization of both the patient and clinician versions of the STAR questionnaire as consisting of three subscales [16, 19], the quality of the TR from the points of view of patients and attending physicians were described as latent variables in SEM (Fig 2).

The three corresponding subscales per latent variable served as observable indicator variables. The results of the SEM analysis are summarized in Fig 2. With a nonsignificant chi-square test and a comparative fit index (CFI) coefficient of. 95, sufficient relative model fit seems to apply, even though the root mean square error of approximation (RMSEA) coefficient of. 08 was only slightly above the threshold considered indicative of sufficient absolute model fit (.07). In the SEM model, all factor loadings were significant at the alpha = .001 level (two-sided). Concerning the hypothesis, coercive treatment as such did not seem to be associated with more negative judgments of the quality of the TR (Table 2): On average, only the ratings of involuntarily admitted general psychiatric patients turned out to be more negative than the ratings of self-referred patients from general psychiatry (*p* = .012, two-sided). The relative size of that difference in mean values indicated a medium-sized effect (*d* = 0.60). On average, the ratings of forensic psychiatric patients on the quality of the TR did not differ from the mean rating of self-referred patients. There was a tendency of physicians in forensic psychiatry to rate the mean quality of the TR as more positive. At a *d* value of 0.22, the size of this effect was weak, however, and statistically nonsignificant (*p* = .055, two-sided). This is contrary to our prior expectation. Furthermore, no agreement in judgments of the quality of the TR between the ratings of patients and physicians could be observed. For all three groups, the correlation between the two latent variables of clinician and patient ratings of the TR turned out to be nonsignificant, at *r* = .08, .07, and −.04 for self-referred, involuntary, and forensic psychiatric patients, respectively. Hence, the relatively low estimates of this association reported in previous studies were likely not attributed to measurement error. The size of the correlation turned out to be about zero in the present SEM model (S1 Supporting Information), which is free of measurement error.

**Table 2:** The mean STAR total and subscale scores for groups of self-referred, involuntary admission, and forensic patients as well as for the whole sample.

	Total sample (*n* = 113)	Self-referred group (*n* = 42)	Involuntary admission group (*n* = 34)	Forensic group (*n* = 37)
	Mean (*SD*)	Mean (*SD*)	Mean (*SD*)	Mean (*SD*)
**STAR-C**				
Subscale “PZ-C” positive collaboration (items 1,2,5,7,10,12)	2.81 (0.58)	2.75 (0.59)	2.72 (0.57)	2.96 (0.57)
Subscale “PE-C” positive clinician input (items 3,8,11)	3.11 (0.54)	3.01 (0.66)	2.98 (0.54)	3.26 (0.34)
Subscale “ES-C” emotional difficulties (items 4,6,9) R*	3.28 (0.48)	3.33 (0.47)	3.21 (0.47)	3.29 (0.50)
Total score	3.00 (0.46)	2.97 (0.48)	2.90 (0.47)	3.11 (0.42)
**STAR-P**				
Subscale “PZ-P” positive collaboration (items 2,3,5,6,8,11)	2.73 (0.94)	2.96 (0.89)	2.37 (1.15)	2.84 (0.67)
Subscale “PE-P” positive clinician input (items 1,10,12)	2.45 (0.94)	2.64 (0.91)	2.13 (1.15)	2.54 (0.60)
Subscale “NE-P” non-supportive clinician input (items 4,7,9) R*	2.68 (0.85)	2.92 (0.82)	2.40 (0.89)	2.66 (0.80)
Total score	2.65 (0.76)	2.87 (0.71)	2.32 (0.91)	2.71 (0.56)

*Reversely coded.

Therefore, we would argue that either the perceptions of patients and clinicians on the quality of the TR differ or that the patient and clinician versions of the STAR questionnaire measure different constructs.

Because the goodness of fit of the SEM model could only be ascertained with limited statistical power (.275) owing to the comparatively small sample size, we repeated the group comparison at the overt level, using a multivariate analysis of variance (MANOVA). The MANOVA results mirrored the SEM results (see Supplementary Material for details).

## Discussion

This study is the first to examine both patients’ and their physicians’ views of their TR in therapeutic settings with varying degrees of legal coercion. We did not find an association between the legal status of patients and the quality of the TR. Consequently, it is unlikely that mandatory treatment would necessarily affect the TR negatively. In fact, in any legal setting, a syndrome of hostility influenced the TR the most.

We also investigated the effects of psychopathological symptom clusters (assessed by BPRS) on the patients’ estimation of the quality of the TR. A significant correlation was observed with hostility and patients’ ratings but not with clinicians’ ratings.

### Therapeutic relationship

As far as the perspectives from which the TR is viewed are concerned, both clinicians and patients showed a positive perception of the TR. Clinicians rated the quality of the TR as slightly better than patients in any setting. Previous studies have reported higher patient, clinician, and corresponding ratings [24–26]. Several studies investigating the agreement between patients and physicians on the quality of the TR have shown inconsistent results [25, 27, 28]. Physician ratings of the TR have been described as positive, for instance, when patients showed higher levels of insight [27, 29] or better social abilities [11, 30]. The different neurocognitive abilities of patients have also been associated with discrepancies in the ratings of the TR: Whereas a poorer performance on verbal memory was associated with better patient ratings of the TR, a better performance on visuospatial reasoning was associated with better therapist ratings of the TR [28]. Because patient and therapist agreement on the quality of the TR is associated with a better treatment outcome [13], it should be an important focus of current development in psychiatry. The interpersonal conduct of clinicians should be simplified to accommodate for patients’ deficits in neurocognitive and psychosocial functioning.

### Symptoms

The lowest ratings of the TR and the highest discrepancy between patients’ and physicians’ ratings were found in patients with high hostility scores. Similar associations between patients’ symptoms and ratings of the TR have been previously described [24, 29, 30]. We found that neither legal detention nor the presence of psychotic symptoms in general was associated with the ratings of the TR. Hostility, however, was clearly associated with the ratings of the TR: The higher the hostility symptom load, the lower the ratings of the TR. Previous research has reported on the association between the hostility of the patient directed at the physician and physician depression [31]. Therefore, finding new strategies to tackle hostility may also contribute to the mental health and well-being of health-care professionals.

## Conclusion

Our findings do not support our initial hypothesis that the quality of the TR is associated with the degree of legal coercion. The patients under a mandatory treatment order in general psychiatry—not the forensic psychiatric patients—on average rated the quality of the TR as significantly worse than did the self-referred patients from general psychiatry. A possible explanation could be that the perceived level of restriction was stronger for the patients who were admitted involuntarily to general psychiatric wards than for forensic psychiatric patients [32, 33], especially since the former had not been hospitalized for as long as the latter. This explanation seems unlikely, however, because treatment duration was unrelated to STAR ratings. In addition, we found the psychopathological symptom load in general and the clinical syndrome of hostility in particular to be associated with the quality of the TR from both the patients’ and the clinicians’ perspectives. These findings reveal an important target for interventions to improve the TR: Psychopharmacological options as well as communicative strategies (e.g., feedback mechanisms) should be applied to tackle the relevant clinical symptoms.

### Strengths and limitations

The return rates of patients’ and clinicians’ questionnaires were 100%. According to several study participants, they appreciated the opportunity to express their opinions, which may have led to the high return rates.

To avoid confounders between type of illness (acute vs. chronic) and demographic aspects, we used two homogenous control groups. Although the total number of patients in the forensic inpatient group (*n* = 37) might be considered too small to make significant generalizations, the analysis yielded statistically significant results. Hence, the effect size of the significant associations was at least moderate and, thus, clinically meaningful. The participants’ group size was predetermined by the number of forensic inpatients with a diagnosis of schizophrenia available in our setting during the examination period. We endeavored to avoid a social desirability bias in the forensic and involuntary treatment groups by making clear that their participation in the study would not have any influence on their legal conditions.

Because hostility is a potential symptom associated with schizophrenia, we treated hostility as an independent variable predicting the quality of the TR. It is conceivable, however, that the relationship between hostility and the quality of the TR is in another direction; that is, a TR of insufficient quality may increase the level of a patient’s hostility, or the two variables may influence each other. These possibilities cannot be resolved in a cross-sectional study design like the present one but could be addressed in a prospective study.

Our participants were exclusively male. Consequently, generalizing our findings to female patients with schizophrenia would be tentative. The focus on male participants is attributed to the gender ratio in forensic psychiatry, where the vast majority (about 95%) of patients is male.

Furthermore, the TR can be viewed as a dynamic construct with high and low periods of intensity over time. Our cross-sectional study did not allow for the quality of the TR to be investigated over time and for bidirectional causality between the factors to be analyzed. Although there was a considerable difference in the average length of stay of general psychiatric patients on the one hand and forensic psychiatric patients on the other, it is unlikely that this difference influenced the quality of the TR as we could not find any correlation between treatment duration and ratings on the quality of the TR. Finally, it would be of interest to triangulate findings of the differences between patients’ and physicians’ perspectives through a more objective examination of the TR from the perspective of a neutral observer who is not involved in the therapeutic process.

The data structure was nested insofar as some physicians rated the TR with respect to several patients. It was not possible, however, to adjust for the nesting statistically because the nesting did not carry over to the second level of the clustering (patient groups): The clinicians who rated several patients did so within patient groups only, not across the different patient groups.

## Supporting Information

S1 Supporting Information SEM Model. Here we describe the structural equation model (SEM) by Kim [33], which was what we used given our relatively small sample size.
(DOCX)
