# Supplementary material for: The Impact of Legal Coercion on the Therapeutic Relationship in Adult Schizophrenia Patients
Source: PLoS One. 2015 Apr 24;10(4):e0124043. doi: 10.1371/journal.pone.0124043 (PMC4409206; doi:10.1371/journal.pone.0124043)
Supplement: S1 Supporting Information — Here we describe the structural equation model (SEM) by Kim [33], which was what we used given our relatively small sample size. (DOCX) [file pone.0124043.s001.docx]

**S1 Supporting Information. SEM Model.**

Based on a comparative fit index (CFI) of 0.95 and using the method of power estimation for structural equation modeling (SEM) by Kim [33], we estimated the achieved power of the SEM model to be only .275. We used the covariance matrix of the reference group (i.e., the self-referred general psychiatric patients) as a basis for the estimation of achieved power. In other words, the measurement model shown in Figure 2 has a conditional probability of about one-fourth only to be recognized as non-fitting, given the sample size (*N* = 113), the degrees of freedom of the model (41), and the type I error rate (alpha = .05). To achieve sufficient levels of power like .80 or .90, sample sizes of 281 or 342 individuals, respectively, would be necessary. For the basic structural model without a mean structure, however, the achieved power was higher (0.50). Consequently, there was a 50% likelihood of recognizing a non-fitting structural model as such.

Therefore, the group differences with regard to estimates of the quality of the TR and the correlations between the patients’ and physicians’ ratings were also analyzed at the observed level, using a multivariate analysis of variance (MANOVA). In MANOVA, patient groups (self-referred vs. involuntary vs. forensic) served as the independent variable. The dependent measures were the total scores on the clinician and patient versions of the STAR questionnaire. Planned contrasts were analyzed as simple contrasts between the reference group of self-referred patients and each other group. A prior Box’s M test remained nonsignificant, indicating that the covariance matrices of the three groups can be considered equal, *F*(6, 250981.07) = 1.47, *p* = .185. Overall, MANOVA turned out to be significant, *F*(4, 220) = 3.61, *p* = .007, at an effect size of partial eta-squared = .06. In univariate terms, the main effect of group turned out to be significant with regard to the patients’ ratings only, *F*(2, 110) = 5.56 (*p* = .005), partial eta-squared = .09. The physicians’ ratings of the quality of the TR (*M* = 2.99, *SD* = 0.46) did not differ between groups to a significant degree, *F*(2, 110) = 2.00, *p* = .141, partial eta-squared = .04. As far as the simple linear contrasts for the patients’ ratings of the quality of the TR are concerned, only the mean differences between the groups of self-referred and involuntary general psychiatric patients turned out to be significant (*p* < .001), with the latter on average giving a lower rating (*M* = 2.32, *SD* = 0.91) than the former. The difference in the mean ratings given by self-referred patients from general psychiatric wards (*M* = 2.87, *SD* = 0.71) and by forensic psychiatric patients (*M* = 2.72, *SD* = 0.56) was statistically nonsignificant (*p* = .355). Hence, the results of MANOVA based on the observed data mirrored the results of the SEM analysis: The only significant difference turned out to be the one between self-referred and involuntary patients within general psychiatry, with involuntary patients on average judging the quality of the TR as lower than self-referred patients.
